# Focal subarachnoid haemorrhage mimicking transient ischaemic attack - do we really need MRI in the acute stage?

**DOI:** 10.1186/1471-2377-14-80

**Published:** 2014-04-10

**Authors:** Lorenz Ertl, Dominik Morhard, Maria Deckert-Schmitz, Jennifer Linn, Gernot Schulte-Altedorneburg

**Affiliations:** 1 Department of Radiology, Nuclear Medicine & Neuroradiology, Klinikum München-Harlaching, Sanatoriumsplatz 2, Munich D-81545, Germany; 2 Department of Neuroradiology, University of Munich, Marchioninistr 15, Munich D-81377, Germany; 3 Department of Neurology, Klinikum München-Harlaching, Sanatoriumsplatz 2, Munich D-81545, Germany

**Keywords:** Transient ischaemic attack, Subarachnoid haemorrhage, Computed tomography, Magnetic resonance imaging, Emergency care

## Abstract

**Background:**

Acute non-traumatic focal subarachnoid haemorrhage (fSAH) is a rare transient ischaemic attack (TIA)-mimic. MRI is considered to be indispensable by some authors in order to avoid misdiagnosis, and subsequent improper therapy. We therefore evaluated the role of CT and MRI in the diagnosis of fSAH patients by comparing our cases to those from the literature.

**Methods:**

From 01/2010 to 12/2012 we retrospectively identified seven patients with transient neurological episodes due to fSAH, who had received unenhanced thin-sliced multiplanar CT and subsequent MRI within 3 days on a 1.5 T scanner. MRI protocol included at least fast-field-echo (FFE), diffusion-weighted imaging (DWI), T2-weighted fluid-attenuated inversion recovery (FLAIR) and time-of-flight (TOF) MRA sequences. By using MRI as gold-standard, we re-evaluated images and data from recent publications regarding the sensitivity to detect fSAH in unenhanced CT.

**Results:**

fSAH was detected by CT and by FFE and FLAIR on MRI in all of our own cases. However, DWI and T2w-spin-echo sequences revealed fSAH in 3 of 7 and 4 of 6 cases respectively. Vascular imaging was negative in all cases. FFE-MRI revealed additional multiple microbleeds and superficial siderosis in 4 of 7 patients and 5 of 7 patients respectively. Including data from recently published literature CT scans delivered positive results for fSAH in 95 of 100 cases (95%), whereas MRI was positive for fSAH in 69 of 69 cases (100%).

**Conclusions:**

Thin-sliced unenhanced CT is a valuable emergency diagnostic tool to rule out intracranial haemorrhage including fSAH in patients with acute transient neurological episodes if immediate MRI is not available. However, MRI work-up is crucial and mandatorily has to be completed within the next 24–72 hours.

## Background

Diagnosis of transient ischemic attack (TIA) and differentiation from TIA-mimics is challenging, since misdiagnosis may lead to inappropriate treatment. Recent trials on TIA patients have shown that clinical scores are of limited use in identifying individuals at risk if neuroimaging is not considered [1].

Several authors have reported a rare but important migraine-like TIA-mimic syndrome consisting of migratory, crescendo-like, somatosensory symptoms with a spontaneous focal subarachnoid haemorrhage (fSAH) of the contralateral cerebral hemisphere [2-8]. Neuroimaging reveals a characteristic pattern of fSAH, mostly in the corresponding pre- or postcentral sulcus.

There is still no standardised diagnostic work-up for fSAH patients, but such a work-up is of great importance, since therapy schemes of acute intracranial haemorrhage and cerebral ischemia differ substantially. Many authors recommend MRI as the first-choice technique to detect fSAH, since CT is suspected to be insensitive to circumscribed subarachnoid haemorrhage [2,3,5,9]. This statement is problematic from a medico-legal point of view for any neurological department participating in TIA care without providing MRI on a 24/7 basis.

In this study, neuroradiological and clinical data of seven of our own patients with fSAH are presented to evaluate the role of CT in comparison to MRI as a primary diagnostic tool. We compared our imaging findings to previously published cases of fSAH.

## Methods

### Subjects

Our institution provides an accredited stroke unit on a 24/7 basis and a TIA outpatient clinic from 8 a.m. to 5 p.m. on weekdays. Standard diagnostic work-up of patients presenting at our hospital with transient neurological episodes is as follows. During the opening hours of our TIA outpatient clinic, immediate MRI examination including FLAIR, DWI, FFE and TOF-MRA sequences is done. If MRI is not available, an unenhanced cranial CT scan and an ultrasound examination of the extra- and intracranial brain-supplying arteries is performed. MRI work-up is completed as soon as possible within 24–72 hours.

Patients were identified by a retrospective query on all cranial MRI examination reports (n = 7482) in the radiological database of our institution between 01/2010 and 12/2012. The following search terms were used: “siderosis”, “subarachnoid hemorrhage”, “focal SAH”. The primary search yielded 243 patients. Patients were included if they fulfilled the following criteria: 1.) presence of a focal SAH at the cerebral convexity, defined as a linear hyperintensity on FLAIR-images and linear hypointensity in T2*-images in MRI; 2.) patients had received an unenhanced CT scan of the brain prior to the MRI.

Patients were excluded if they had obvious causes of bleeding such as 1.) aneurysmal SAH or SAH from other intracranial vascular malformations (n = 45), 2.) traumatic SAH (n = 45), 3.) primary intracerebral bleeding or haemorrhagic brain tumor with extension to the subarachnoid space (n = 25), 4.) derailment of blood coagulation (n = 3).

Finally, we identified seven patients with non-traumatic fSAH during this three year period who met the inclusion and exclusion criteria (Table 1).

**Table 1:** **Patients**’ **clinical and radiological characteristics**

**Pat. no.**	**Symptoms**	**Episodes (duration)**	**Localization fSAH**	**Delay last episode to CT/MRI**	**CT positive**	**MRI**			**EEG**
						**fSAH positive**	**SS**	**MB**	
1	Left-sided sensory deficits	>1 (5–15 min)	Right precentral	<24 h / <24 h	Yes	FLAIR, FFE, T1w+/-Gd	None	None	Normal
2	Aphasia, numbness (right upper extremity)	2 (10–30 min)	Left precentral	<24 h / <24 h	Yes	FLAIR, FFE,DWI, T1w+/-Gd	Bilateral frontoparietal	None	Theta patterns left occipital
3	Aphasia, psychomotor deficits, spreading hypaesthesia (right hand)	>1 (10–30 min)	Left central	<24 h / <48 h	Yes	FLAIR,FFE, T1w+/-Gd	Front partial & pontine	12	Alpha pattern (8/s) delta decelerations left parietotemporal
4	Left-sided paraesthesia	>1 (5–10 min)	Right central	2d /5d	Yes	FLAIR,FFE, T2w,T1w	Bilateral frontal	18	Normal
5	Vertigo, fluctuating left-sided sensomotory deficit	4 (10-30 min)	Right central	<24 h / <48 h	Yes	FLAIR,FFE, T2w,DWI, T1w+/-Gd	None	>20	n.a.
6	Acute cognitive deficits, mild right-sided hypaesthesia	>1 (5–10 min)	Left precentral	<24 h / <48 h	Yes	FLAIR,FFE, T2w,T1w + Gd	Bilateral frontoparietal	>30	Slowed baseline activity, intermittent generalized decelerations
7	Hypaesthesia (left face/hand)	3 (5 min)	Right central	<24 h / <24 h	Yes	FLAIR,FFE, T2w,DWI, T1w+/-Gd	Bilateral frontoparietal & temporal	None	Normal

After identification of the patients, demographic and clinical characteristics and the final diagnosis were retrieved from hospital records (Table 1). An experienced stroke neurologist (M.S.-D.) verified all clinical symptoms and diagnoses, including clinical history, electroencephalogram (EEG), electrocardiogram, echocardiography and routine blood tests.

### MR imaging

All MRI examinations were performed on a 1.5 T scanner (Intera, 1.5 T, Philips GmbH-Healthcare, Hamburg, Germany). In all cases imaging protocol included fast-field-echo (FFE), diffusion-weighted imaging (DWI), T2-weighted fluid-attenuated inversion recovery (FLAIR), T2-weighted spin-echo sequence, and time-of-flight MR angiography (TOF-MRA) of the intracranial arteries. In five patients, T1-weighted unenhanced and contrast-enhanced (0.1 mmol Gd-DTPA per kg body weight) scans were acquired (pat. 1, 2, 3, 6, 7). Phase-contrast (PC)-MRA of the cerebral veins and sinuses was obtained in six patients (pat. 1, 2, 4–7). Microbleeds (MB) were identified and differentiated from intracerebral haemorrhage according to Greenberg et al. [10]. Superficial siderosis (SS) was defined as linear blood residues in several superficial cortical layers of the brain on FFE images. fSAH was differentiated from SS, when it was an isolated finding in a neuroanatomical location corresponding to the TIA-like symptoms.

### Computed tomography

**Figure 1:**
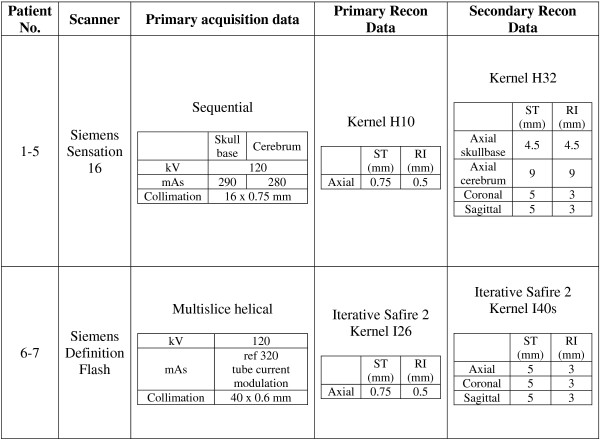
Physical acquisition parameters of CT scanner.

Axial multislice CT scans were obtained on a 16-slice or a dual-source 2x128-slice scanner (Somatom Sensation 16 and Definition FLASH, both Siemens Healthcare, Forchheim, Germany) in helical-scan mode using 0.6-0.75 mm collimation. Two sets of reconstructions with 0.75/0.5 mm and 4.5-9/3-5 mm (slice-thickness/increment) were calculated. The detailed protocols of CT scanning and multiplanar reconstructions, including reconstruction interval, collimation and slice thickness, are given in Figure 1.

### Additional vascular imaging

All patients underwent extracranial cervical doppler sonography. Conventional four-vessel angiography was performed in two patients (patients 1 and 3), whereas cervical contrast enhanced MRA was available in two cases (patients 1 and 7). Patient 4 underwent intra- & extracranial CTA.

### Image analysis

Neuroradiological images were re-evaluated by three experienced neuroradiologists (L.E., D.M., G.S.-D.). Prior to the reading process, all individual patient data in the images were replaced by a pseudonomized numeric code. CT and MR images were evaluated in a randomised order. Thus, readers were blinded to the individual CT and MR examinations as well as to the clinical history. After the individual reading process, the participating radiologists met for a consensus reading, leading to a common interpretation of the imaging material. Venous and arterial MRA, CTA and conventional angiographies were also re-analysed with special regard to vascular malformations, vasculitides and cortical vein thrombosis. Due to the retrospective study design, institutional review board approval was not needed.

### Literature search strategy

Articles were identified in the PubMed-Medline database up to December 2013. The following key words were used for the search: “focal/non traumatic/cortical subarachnoid haemorrhage”, “transient ischaemic attack”, “microbleeds”, “cerebral amyloid angiopathy”, “crescendo aura attacks” (limits were English and humans).

Publications were selected if they reported on radiological and clinical characteristics of fSAH including CT and MRI. Additionally, a manual search of references was also performed.

## Results

### Own data

fSAH was detectable in primary unenhanced CT scan in all of 7 cases. Clinical and radiological characteristics are given in Table 1. All patients (5 female, 2 male) were older than 67 years and suffered predominantly from sensory deficits. In 6 of 7 patients, the delay between the last episode and the CT examination was < 24 hours. In none of the cases vascular imaging revealed a stenosis of > 50% lumen reduction or showed evidence for vasculitis, RCVS or any other vascular malformation. All patients were discharged without recurrence of symptoms.

### Literature research

Eight case series were identified with a total of 102 patients, including our own data (Table 2). 100 of 102 patients underwent an unenhanced CT scan that delivered positive results for fSAH in 95 cases (95%). MRI was available in 97 of 102 patients. 28 of those were found in a publication in which the exact number of positive findings for fSAH in MRI was not provided [8]. They were thus excluded, resulting in a total of 69 MRI examinations, of which 69 were positive for fSAH (100%).

**Table 2:** fSAH in recent literature compared with own data

**Ref. no.**	**Number of patients**	**Mean age (range)**	**CT positive for fSAH**	**MRI positive for fSAH**	**Percentage [Pos CT/Pos MRI]**
[2]	7	74 (63–82)	5/7	7/7	70%
[3]	4	78 (68–85)	4/4	4/4	100%
[4]	2	74 (73–74)	n.a.	2/2	-
[5]	10	74 (62–87)	10/10	10/10	100%
[6]	2	76 (70–81)	2/2	2/2	100%
[7]	24	70 (37–88)	24/24	20/20	-
[8]	29	58 (29–87)	28/29	n.a./28	-
[15]	17	78 (69–96)	15/17	17/17	88%
*Own data*	7	79 (68– 84)	7/7	7/7	100%
*Total*	102	-	95/100 (95%)	69/69(100%)	-

## Discussion

During a three-year period, we detected seven cases of TIA-mimicking non-traumatic fSAH in a study population of subjects who had received unenhanced CT and MR imaging work-up. fSAH was detectable in both modalities in all of the cases. This is in line with previously published cases from the literature (Table 2).

The clinical symptoms and radiological appearance of fSAH differ completely from those of SAH following the rupture of an aneurysm or non-aneurysmal perimesencephalic SAH [2,3,5,9]. Patients experience recurrent, transient, spreading somatosensory deficits lasting a few to several minutes. An associated headache is usually not found in elderly patients [8].

Several aetiologies have been found to be associated with fSAH [8,11]. According to Kumar, the most probable cause of fSAH in patients older than 60 years is cerebral amyloid angiopathy (CAA), while reversible cerebral vasoconstriction syndrome (RCVS) is the most common aetiology in patients younger than 60 years [8]. Geraldes et al. found large artery atherosclerosis to be the most probable underlying cause in one third of fSAH patients [11]. Similar to Raposo et al. [5], we found preexisting multifocal haemorrhagic lesions (MB and/or SS) in 4 of seven patients. In accordance with previous reports [2,5] our patients were significantly older than patients with the common causes of SAH (i.e. traumatic, aneurysmal).

In the AHA/ASA Scientific Statement, MRI is recommended as superior to CT for managing TIA [12]. In previous TIA studies, however, MRI was frequently the first-line imaging in less than 30%-50% of TIA patients [12], since MRI cannot be tolerated or is contraindicated in ~10% of patients, and 24/7 availability is sometimes not given even in many neurovascular centers.

One of the most difficult problems that clinicians face in patients with transient neurological deficits, is to avoid misdiagnosis of intracranial haemorrhage as a classical TIA and subsequent mistreatment with antiplatelets or anticoagulants. While early antithrombotic therapy is beneficial in patients with TIA, AHA Stroke Council guidelines recommend “individual assessment of each case” in TIA patients with suspected ICH [13]. The authors cite a “paucity of data from large, prospective, randomized studies” to answer this important management question and emphasize the small number of case series addressing SAH as a potential cause of a TIA-mimic [13].

Currently, there is no standardised MRI protocol in TIA patients. Previous studies showing superiority of MRI in TIA patients reported only on the diagnostic value of DWI [12]. However, detection of SAH requires an appropriate MRI protocol that goes beyond the “fast DWI protocol” for stroke and TIA patients. FLAIR sequence is very sensitive to pathologies of the subarachnoid space, but not specific for SAH [14]. GRE or susceptibility-weighted sequences are very sensitive to haemosiderin and therefore important for the diagnosis of fSAH. However, GRE images do not allow differentiation between acute haemorrhage and residual posthaemorrhagic haemosiderin deposits.

Unenhanced CT scans allowed detection of fSAH in all our patients. In accordance with our results, a review of the literature for fSAH revealed that the initial unenhanced CT scan was positive in 95 of 100 cases (95%) (Table 2).

Brunot and co-workers [2] reported 2 of 7 fSAH patients presenting with negative initial CT scans prior to MRI. The authors concluded that fSAH were not systematically detectable by unenhanced CT scans [2]. However, in these two cases, negative CT scans were acquired immediately after the first of several recurrent transient neurological episodes. MRI was performed 3–4 weeks later and after at least one more neurological event [2]. Besides that exact CT acquisition parameters such as collimation were not provided.

Similar to this 2 out of 17 CT scans were negative for fSAH compared to MRI in the series of Apoil et al., but also in this publication no detailed information on timing and physical acquisition parameters of the CT examination was given [15].

The discrepancy between our results and those in the literature might reflect differences in timing and choice of investigations, which indeed is a notable aspect and might limit our findings. We strongly recommend careful analysis of thin-sliced multiplanar primary reconstruction CT images (ST 0.75 mm/RI 0.5 mm) to enhance investigator-dependent sensitivity for fSAH, and completion of MRI work-up within 3 days.

Other authors also recommended MRI as the first choice for diagnosis of fSAH, although they reported a 100% rate of CT in detecting fSAH (Table 2) [3,5,9]. Unfortunately, a detailed comparison of the diagnostic accuracy of CT and MRI was not available in the case series of Kumar et al. [8].

According to our data, careful analysis of multiplanar thin-sliced CT allows for detection of acute fSAH with sufficient diagnostic power in an emergency setting. However, despite its appropriateness as a readily available primary diagnostic tool, CT does not provide important additional information about other manifestations of cerebral small vessel diseases, including microbleeds, siderosis, ischaemic brain injury and newer small vessel disease markers. We thus emphasize the need for an extensive secondary MRI work-up which seems to be crucial for assessment of the likely future risk of ICH [16].

The small number of cases acquired and the retrospective design of our study limits general recommendations from our data and does not allow a statistically valuable sensitivity/specificity analysis, as in previously published works [2,3,5,9,15]. Due to the short period between clinical onset and CT examination, we might have not missed one of the fSAH in CT. Unlike previous studies, our patients (except pat. no. 4) suffered from an acute transient focal neurological episode within 24 hours prior to the CT. In patients, whose last neurological episode dates back more than 48 hour, MRI should be preferred as primary diagnostic tool. Nevertheless, our data as well as the reported data from the literature might encourage stroke-neurologists as well as neuroradiologists to regard CT as a valuable diagnostic tool which allows for recognising even fSAH in TIA-patients with a quite high accuracy in an emergency setting.

## Conclusion

Unenhanced CT is a valuable emergency diagnostic tool to rule out acute intracranial haemorrhage including fSAH in patients with transient neurological episodes if immediate MRI is not available. However, we emphasize that a single unenhanced CT scan cannot be considered sufficient to investigate transient neurological episodes and to guide antithrombotic use on its own. MRI work-up is indispensable for the further detection of SS, MB, and other parenchymal abnormalities and has to be completed within the next 24–72 hours. It should contain at least FLAIR, DWI, FFE and TOF-MRA sequences. Non-invasive vascular imaging is helpful to exclude potential associated, coincidental and additional pathologies. Catheter angiography is then usually expendable.

## Competing interests

The authors declare that they have no competing interests.

## Authors’ contributions

LE and DM carried out the data collection, participated in the image analysis and drafted the manuscript. MDS participated in the data collection, revised all clinical data and helped to draft the manuscript. JL helped to draft the manuscript and participated in the design of the study. GSA designed the study, participated in the data collection and the image analysis and helped to draft the manuscript. All authors read and approved the final manuscript.

## Pre-publication history

The pre-publication history for this paper can be accessed here:

http://www.biomedcentral.com/1471-2377/14/80/prepub
